# Tumoral inflammatory infiltrate does not predict metastases in thin primary cutaneous melanomas^☆^

**DOI:** 10.1016/j.abd.2022.09.011

**Published:** 2023-06-22

**Authors:** Mariele Bevilaqua, Maria Carolina Widholzer Rey, Gian Carlo Antonini Cappellini, Felice Riccardi, Cristina Fortes, Adriana Vial Roehe, Renan Rangel Bonamigo

**Affiliations:** a Department of Dermatology, Universidade Federal de Ciências da Saúde de Porto Alegre, Porto Alegre, RS, Brazil; b Universidade Luterana do Brasil, Canoas, RS, Brazil; c Department of Dermatology, Santa Casa de Misericórdia de Porto Alegre, Porto Alegre, RS, Brazil; d Department of Oncology, Hospital Sandro Pertini, Rome, Italy; e Department of Oncological Surgery, Hospital Santa Rita, Santa Casa de Misericórdia de Porto Alegre, Porto Alegre, RS, Brazil; f Department of Epidemiology, Istituto Dermopatico dell'Immacolata, Rome, Italy; g Department of Patology, Universidade Federal de Ciências da Saúde de Porto Alegre, Porto Alegre, RS, Brazil; h Department of Dermatology, Universidade Federal do Rio Grande do Sul, Porto Alegre, RS, Brazil

**Keywords:** Biomarkers, Lymphocytes, tumor-infiltrating, Melanoma, Neoplasm metastasis, Prognosis

## Abstract

**Background:**

In cutaneous melanomas in general, tumor inflammatory infiltrate (TII) can protect against distant metastases, but there is no consensus when only thin primary cutaneous melanomas (TPCM) are considered.

**Objective:**

To investigate the presence of TII in TPCM and the relationship between TII and the occurrence of metastases.

**Methods:**

Case-control study including 50 patients with TPCM, 22 metastatic (MC group) and 28 non-metastatic (NMC group). The presence of TII was evaluated and, if present, qualified as mild, moderate or marked.

**Results:**

The mean age was 50.7 years in the MC and 56.2 years in the NMC group (p = 0.234), and the male sex predominated in the MC group (63.6%). The average Breslow thickness was higher in the MC when compared to that observed in the NMC (respectively 0.8 vs. 0.6 mm, p = 0.012). The presence of ulceration occurred in 22.7% of the MC and 17.9% of the NMC (p = 0.732). TII was present in all 50 TPCM, being marked or moderate in 67.9% of the NMC and 54.5% in the MC group (p = 0.503). In the multivariate analysis, the presence of moderate and marked TII had an Odds Ratio (OR) of 0.57 (95% Confidence Interval [CI]: 0.18‒1.8) and adjusted OR of 0.68 (95% CI 0.13‒3.99).

**Study limitations:**

Small sample size.

**Conclusions:**

TII was present in all TPCM (with and without metastases), and it was not possible to demonstrate a protective effect of TII against the appearance of metastases.

## Introduction

Primary cutaneous melanoma is the most lethal form of skin cancer, accounting for approximately 90% of deaths related to cutaneous tumors.^1^ The incidence of this malignant neoplasm continues to rise, with approximately 232,000 new cases per year worldwide, accounting for 7% of all diagnosed malignancies, except non-melanoma skin cancer.^1^

Thin primary cutaneous melanomas (TPCM) are those with a Breslow thickness ≤ 1 mm.^2^ Studies show that periodic dermatological evaluations and the increasingly accurate use of the dermoscope have favored an earlier detection of TPCM and in situ melanomas.^3^, ^4^, ^5^, ^6^

The mortality rate from melanoma is known to be strongly associated with the primary lesion thickness (Breslow thickness).^2^ Currently, most new cases of cutaneous melanoma are diagnosed as TPCM, with approximately 90% being diagnosed as primary tumors with no evidence of metastases at the initial approach, according to Aitken et al.^3^ In fact, there is a diversity of statistical data regarding the presence of metastases in the follow-up of TPCM, with studies reporting incidences of 3% to 5% and up to 20% of distant metastasis in TPCM.^7^, ^8^, ^9^

This biological behavior consequently results in scenarios with an unfavorable prognosis.^10^, ^11^ Gimotty et al.^2^ indicate that 15% of deaths from melanoma result from metastases of thin lesions. Specific factors for identifying patients with TPCM at risk for metastases have not yet been fully elucidated.^12^ Many details of the biological behavior and histopathological presentation of metastatic TPCM are partially unknown.

Lymphocytes and mononuclear cells associated with human cancer have been described for more than a century.^13^ Wallace Clark used the term “tumor lymphocytic infiltration” to refer to the immune cells present in the inflammatory infiltrate as part of the host response to cancer.^14^ Regarding cutaneous melanomas, the presence of infiltrating lymphocytes is believed to be associated with a favorable patient outcome, although this is a controversial topic.

Research on the influence of lymphocyte infiltration, specifically on TPCM, is scarce, and the understanding of the phenomenon is incipient. Therefore, the present study investigated the presence of tumor inflammatory infiltrate (TII) in TPCM and the relationship between TII and the occurrence of metastases.

## Methods

### Study population and design

This retrospective, case-control study was carried out in two centers (one in Italy and the other in southern Brazil), based on data found in the records of the Pathology Services of Istituto Dermopatico dell'Immacolata (Rome, Italy) and Irmandade Santa Casa de Misericórdia (Porto Alegre, Brazil), during a two-year period (2017‒2019). The histopathological evaluation was conducted by the Pathology Service of both centers. All histopathological slides were evaluated again by a third specialist in pathology (AVR) from Universidade Federal de Ciências da Saúde de Porto Alegre, who defined the final histopathological diagnosis.

The following clinical and histopathological variables were observed: sex, melanoma topography, metastasis site, the time between melanoma diagnosis and detection of metastases, melanoma subtype, Breslow thickness, growth phase, presence of ulceration, tumor regression, and presence of TII.

TII interpretation followed the definitions used by Fortes et al.^15^:

• Grade 1 (mild): few lymphocytes infiltrate the tumor focally or multifocally along or at the base of the dermal component (≤30% of the component was infiltrated by lymphocytes);
• Grade 2 (moderate): Moderate amounts of lymphocytes infiltrate the tumor focally or multifocally, but not along the entire base of the dermal component (31% to 64% of the component was infiltrated by lymphocytes);
• Grade 3 (marked): a continuous band of lymphocytes was present at the base of or along the melanoma dermal component (≥65% of the component was infiltrated by lymphocytes).

For analysis purposes, cases of moderate and marked TII were added, which were then compared to cases of mild TII.

### Ethical aspects

The study followed the protocols determined by the Ethics Committee of the institution and was approved under number 12447213.8.0000.5345. The Free and Informed Consent Form was signed by all patients or their legal guardians. The authors signed a document agreeing to preserve patient anonymity regarding the use of their data.

### Statistical analysis

Quantitative data were presented as mean and standard deviation. In the case of data asymmetry, the median, minimum and maximum values were used. Categorical variables were counted and transformed into percentages. Student t-test or its non-parametric counterpart (Mann-Whitney test) was used to compare quantitative data. The Chi-square test or Fischer exact test was used for proportions when necessary. Adjustments for possible confounding effects were performed using logistic regression. The significance level was set at p < 0.05. The data were analyzed using SPSS software, version 22.0.

## Results

The present study included 50 patients, 22 of which with metastatic TPCM (cutaneous metastatic, CM group) and 28 patients with non-metastatic TPCM (cutaneous non-metastatic, CNM group). The mean age was 50.7 years in cases (CM group) and 56.2 years in controls (CNM group; p = 0.234). The male sex predominated in the CM group (63.6%) and the female sex in the CNM group (53.6%; p = 0.354). The trunk was the main tumor site of the primary tumor in both groups (p = 0.187), and metastases occurred mainly through hematogenous spread, affecting the lung in nine cases (39.1%) in the CM group.

The most frequent tumor subtype was superficial spreading, observed in 86.4% of the CM and 92.9% of the CNM (p = 0.499) groups. Mean Breslow thickness was 0.8 mm and 0.6 mm, respectively in the CM and CNM groups (p = 0.012), and the presence of ulceration was evident in 22.7% of the CM and 17.9% of CNM groups (p = 0.732). Histopathological regression was observed in 45% of the CM and in 28.6% of the CNM tumors (p = 0.386). Table 1 shows the general data of the sample.

**Table 1:** Profile of patient sample with thin primary cutaneous melanoma (n = 50)

Clinical and histopathological variables	Metastatic (n = 22)	Non-metastatic (n = 28)	p
Male sex, n (%)	14 (63.6)	13 (46.4)	0.354
Age at diagnosis (mean ± SD)	50.7 ± 18.6	56.2 ± 11.4	0.234
Initial tumor site, n (%)			
Head	0/21 (0)	4/28 (14.3)	0.187
Trunk	14/21 (66.7)	15/28 (53.6)	
Limbs	07/21 (33.3)	09/28 (32.1)	
Metastatic spread, n (%)			
Hematogenous	13 (56.5)		
Lymphatic	5 (21.7)		
Both	5 (21.7)		
Melanoma subtype			
Superficial spreading	19 (86.4)	26 (92.9)	0.499
Acral	02 (9.1)	02 (7.1)	
Others	01 (4.5)	0 (0)	
Breslow thickness (mm)	0.8 (0.2‒1)	0.6 (0.2‒1)	0.012
Vertical growth phase, n (%)	12/15 (80)^a^	14/28 (50)	0.012
Ulceration, n (%)	05 (22.7)	05 (17.9)	0.732
Regression, n (%)	09/20 (45)	8/28 (28.6)	0.386

a Growth phase not assessable in seven cases.

**Figure 1:**
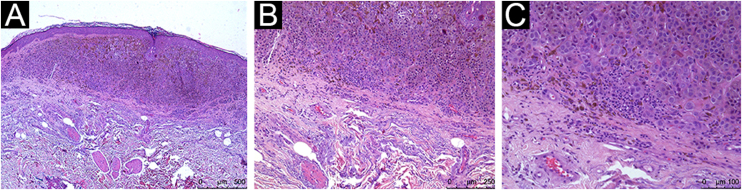
Histopathological analysis showing mild peritumoral inflammatory infiltrate: Hematoxylin & eosin; (A), ×4 magnification; (B), ×10 magnification; (C), ×20 magnification

**Figure 2:**
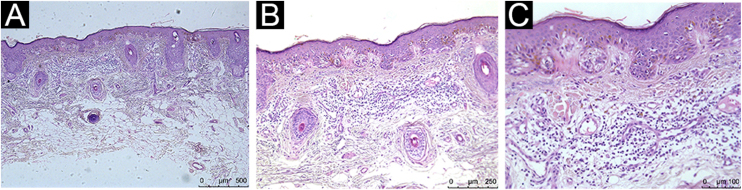
Histopathological analysis showing moderate peritumoral inflammatory infiltrate: Hematoxylin & eosin; (A), ×4 magnification; (B), ×10 magnification; (C), ×20 magnification

**Figure 3:**
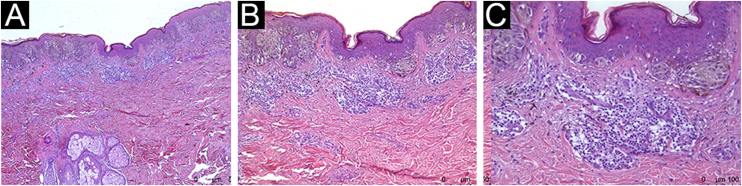
Histopathological analysis showing marked peritumoral inflammatory infiltrate: Hematoxylin & eosin; (A), ×4 magnification; (B), ×10 magnification; (C), ×20 magnification

All assessed patients had TII, with different qualitative and quantitative classifications, with moderate to marked TII being present in 54.5% of the CM and 68% of the CNM tumors. For the crude data, an Odds Ratio (OR) of 0.57 (95% CI 0.18‒1.8; p = 0.338) was obtained (Table 2). The multivariate analysis with adjustments for Breslow thickness, sex and age at diagnosis of melanoma was applied, and the presence of moderate to marked TII showed an OR of 0.68 (95% CI 0.13‒3.99; p = 0.669; Table 2). Figure 1, Figure 2, Figure 3 show histopathological images of mild, moderate and marked TII in patients with TPCM.

**Table 2:** Relationship between tumor inflammatory infiltrate (TII) and occurrence of metastases in thin primary cutaneous melanoma (TPCM)

Characteristic	Metastatic TPCM (n = 22)	Non-metastatic MCPF (n = 28)	Odds ratio (95%CI)
Marked to moderate TII	12 (54.5%)	19 (67.9%)	0.57 (0.18‒1.80); p = 0.338
			0.68 ( 0.13‒3.99); p = 0.669^a^

a Adjusted by multivariate analysis for Breslow thickness, sex, patient age, and tumor growth phase.

## Discussion

The role of the immune response in cutaneous melanomas has gained attention in recent years, particularly with the advent of immunomodulatory therapies for advanced diseases.^16^ TII comprises a heterogeneous group of lymphocytes that differ in their ability to boost the immune system in their anti-tumor capacity.^14^

Several studies have correlated better survival in cutaneous melanoma with the presence of TII, particularly dense (marked) infiltrates. Clark et al.^17^ demonstrated that TII intensity is associated with disease-free survival, and Clemente et al.^18^ found a five-year survival rate of 77% in patients with dense TII, compared with 53% in those with non-dense TII and 37% in patients without TII. Tuthill et al.^19^ showed a survival rate of 100% after five years and 93% after ten years in patients with dense TII.

However, some studies have not disclosed a significant association between TII and survival rate in patients with melanoma.^14^, ^20^ These results may be due to the inclusion of data on TPCM that are in the radial growth phase, as during this stage, there is little support for considering TII as an outcome predictor, or due to the misclassification of lymphocytes found near the tumor as being TII.^21^

In the present study, the clinical and histopathological variables were in line with findings from previous studies.^7^, ^22^, ^23^ There was a high frequency of vertical growth phase melanomas in patients with metastases compared to those without malignant spread (p = 0.012), with higher Breslow thickness found in those affected by metastases (p = 0.012). Regarding topography, the highest occurrence of melanoma was on the trunk, and the most frequently found subtype was superficial spreading melanoma. The male sex was more often affected among patients with metastases (63.6%). The presence of ulceration is rarely found in TPCM, but when present, it changes cancer staging from T1a to T1b (according to the American Joint Committee on Cancer).^24^ In the present study, no differences were observed in the presence of ulceration between the two groups. Tumor regression on histopathology occurs in 10% to 35% of cutaneous melanomas.^15^, ^25^ In the present study, it was more frequently present in individuals with metastases (45% vs. 28.6%; p = 0.386). A recent multicenter study^26^ reported that regression is highly correlated with TII, but only TII is significantly associated with lymph node metastases and overall survival in patients with melanoma (regression alone was not associated with a different outcome).

Even admitting the low incidence of metastases in patients with TPCM, when compared to invasive melanomas, there are controversies in the literature regarding the data. These differences may be related to the diagnostic phase of the primary tumor, or to other factors of the individual and the bioimmunological characteristics of certain tumors.^7^, ^8^

The data of the present study could not demonstrate TII protection against the development of metastases, as all the assessed patients, with and without metastases, had the infiltrate. Although patients with non-metastatic TPCM more frequently exhibited moderate to marked TII when compared to metastatic patients – 67.9% vs. 54.5%; respectively – no statistical significance was demonstrated (OR = 0.68; p = 0.669).

The presence of TII in all patients with thin melanomas (either metastatic or not) in the present study may indicate that the role of lymphocytic infiltrates is to try to prevent the melanoma from progressing to the phase of greater invasion early, even if it cannot prevent the metastatic spread of a few TPCM.

The limitation of the present study is due to the low frequency of occurrence of metastatic cases in TPCM, with an impact on the reduced sample size for carrying out the evaluation. On the other hand, the fact that this study found variables already established in the literature regarding melanoma characterization, including prognostic factors such as Breslow thickness and the vertical growth phase, demonstrates the adequate quality of the obtained data.

Studies on TII are increasingly necessary, given the known importance of the immune system in determining cancer behavior and the emerging role of inhibiting immunological control in melanoma treatment. To provide more consistent results, it is suggested that longitudinal studies be conducted with a larger sample size. As one of the perspectives, the characterization of the TII immunophenotype can provide relevant information. Finally, it is important to emphasize that, before being adopted in clinical practice, tumor biomarkers must have clinical and analytical validation, as well as a demonstration of clinical usefulness.^27^

## Conclusion

All patients with TPCM had TII, and there were no detectable statistical differences between the metastatic and non-metastatic groups, although the frequency of marked to moderate TII occurred mainly in non-metastatic TPCM.

## Financial support

This research did not receive any specific grant from funding agencies in the public, commercial, or not-for-profit sectors.

## Authors' contributions

Mariele Bevilaqua: Design and planning of the study; data collection; analysis or interpretation of data; statistical analysis, drafting and editing of the manuscript; critical review of the content; approval of the final version of the manuscript.

Maria Carolina Rey: Data collection; analysis or interpretation of data; approval of the final version of the manuscript.

Gian Carlo Antonini Cappellini: Data collection; analysis or interpretation of data; critical review of the content; approval of the final version of the manuscript.

Felice Riccardi: Data collection; critical review of the content; approval of the final version of the manuscript.

Cristina Fortes: Data collection; critical review of the content; approval of the final version of the manuscript.

Adriana Vial Roehe: Data collection; analysis or interpretation of data; critical review of the content; approval of the final version of the manuscript.

Renan Rangel Bonamigo: Design and planning of the study; drafting and editing of the manuscript; critical review of the content; effective participation in research orientation; approval of the final version of the manuscript.

## Conflicts of interest

None declared.
